# State Medical Association of Texas, Thirty-fifth Annual Session, San Antonio, Texas

**Published:** 1903-05

**Authors:** 


### Society Notes.

### State Medical Association of Texas, Thirty-fifth Annual Session, San Antonio, Texas.

The 35th Annual Session of the State Medical Association of Texas was held at Turner's Hall, San Antonio, April 28th, 29th, 30th and May 1st. About 200 physicians registered, but less than half that number participated in the program at any time. The attendance daily fell off till a mere handful were present the last day. This was a surprise and a disappointment, in as much as it was hoped that a renewed interest would be awakened, now that a new constitution has been adopted under which it is hoped to organize the whole profession of Texas. It was due, I think, to the outside attractions; San Antonio, being a historic city, has much to interest visitors and sight-seers.

The Association was called to order by Dr. J. S. Lankford, vice chairman of the Committee on Arrangements.

An invocation was offered by Rev. Arthur G. Jones, pastor First Presbyterian Church of this city.

Hon. J. E. Webb, representing the San Antonio municipality, welcomed the members of the Association to the city.

Hon. Wm. Aubrey welcomed the Association on the part of the citizens of San Antonio.

Dr. Frank Paschal, on behalf of the medical profession of San Antonio, welcomed the Association as follows:

"*Ladies, and Colleagues of the Texas Medical Association:*

On behalf of the medical profession of this city I extend you a cordial welcome. You are always welcome. We feel that you made no mistake in selecting San Antonio as your place of meeting. One word as regards the profession of this city, which I have the honor to represent in extending to you this welcome. It was here that chloroform was administered for the first time in the history of the State. It was here that the first hysterectomy was done. It was here that the first ovarian section was done. It was here that the first lithotomy was done. We have just completed a magnificent hospital in this city at a cost of $50,000, every cent of the capital stock being paid. I feel that in welcoming you to this city we are fully prepared to entertain you and I sincerely hope and trust that we shall always be in unity and harmony with each other, and I hope that you will be so pleased with our city, and with us, that you will come again."

Dr. Red, president of the Association, responded to Dr. Paschal's address in the following manner:

"It is with unfeigned pleasure that I bid you welcome on behalf of the medical fraternity. We have been here before and partaken of your hospitality and explored your beautiful city. I can assure you that we will not carry away the Alamo. You have done everything to make us enjoy ourselves and we have come prepared to do so. Everybody knows of the peculiar flavor of the hospitality of San Antonio. This hospitality seems to surround everything in San Antonio."

The roll was called and a quorum being found present the minutes of the last meeting were read and approved.

Dr. H. A. West, secretary, read his annual report, which, on motion, was referred to a committee of three appointed by the president, consisting of Drs. T. J. Bell, W. Shropshire and R. E. L. Miller.

Dr. R. F. Miller, treasurer, read his annual report, which, on motion of Dr. West, was referred to the same committtee as that of the secretary.

Dr. Cantrell presiding, the president, Dr. S. C. Red, read his annual message and recommendations, which, on motion of Dr. M. M. Smith, were received and referred to a committee of three, composed of Drs. M. M. Smith, Taylor Hudson and J. D. Osborn.

Dr. J. N. McCormack, of Bowling Green, Kentucky, delivered an address on organization. The Association gave a vote of thanks to Dr. McCormack for his valuable address.

Dr. Osborne moved that Dr. McCormack's name be sent to the judicial council for membership on the honorary list. Carried.

On motion, an adjournment was had until 2:30 p. m.

THE AFTERNOON SESSION.

The Association was called to order at 2:30 p. m. by the president, Dr. Red.

Dr. James H. Bell moved that a recess be taken for thirty minutes in order that the delegates might have time to register.

After a resolution of sympathy for Dr. Wilson, of Sherman, who is very ill, had been offered, a resolution was passed, upon motion of Dr. Moore, extending the courtesies of the Association to Dr. Stiles, as representative of the Marine Hospital Service.

The executive session was adjourned, and Dr. J. T. Moore, of Galveston, as chairman of the section on general medicine, called that section to order and read his annual address.

Dr. Love, of New York City, not being present to read a paper on "Medical Side of Surgical Cases," Dr. J. S. Lankford, of this city, read a paper entitled "Diphtheritic Paralysis." The paper was discussed by Drs. W. A. Harper, of Austin; W. B. Collins, of Lovelady, and C. W. Goddard, of Holland.

Major Charles F. Mason, U. S. A., Fort Sam Houston, read a paper entitled "Malta Fever—Report of a Case." The paper was discussed by Drs. H. W. Crouse, of Victoria; H. A. West, of Galveston; Wm. Keiller, of Galveston, and J. T. Moore, of Galveston.

Dr. H. A. West, of Galveston, read a paper, the title of which was "Some Remarks on the Subjective Symptomatology of Heart Disease." Discussion was indulged in by Drs. H. W. Crouse, of Victoria; Frank Paschal, of San Antonio; E. W. Link, of Palestine, and W. B. Collins, of Lovelady.

These papers closed the section on general medicine, and the section on obstetrics and diseases of children was then called.

The chairman of that section, Dr. J. C. Anderson, of Granger, being engaged in important committee work, Dr. H. A. West moved that Dr. J. T. Moore be continued as chairman of the section Carried.

The papers of Drs. J. C. Anderson and E. M. Anderson were passed, and Dr. H. W. Crouse, of Victoria, read a paper on "Circular Laceration of the Cervix Uteri."

Dr. John B. Holt, of Lockhart, read a paper entitled "Bronchitis in Children." Discussion followed by Drs. Crouse, of Victoria; Bethel Nowlin, of Jonah; W. C. Moore, of Runge; Frank Paschal, of San Antonio, and William Keiller, of Galveston.

Dr. J. C. Anderson, of Granger, having completed his committee work, assumed charge of the section on obstetrics and read a paper entitled "Some Facts Concerning the History and Development of the Obstetric Art."

On motion of Dr. Wm. Keiller, of Galveston, the Association adjourned until 9 o'clock Wednesday.

BARBECUE AT HOT WELLS.

The Association adjourned a few minutes before 6 p. m. and all members hurried to the corner of Houston and Navarro streets, where special cars awaited, furnished complimentary by the San Antonio Traction Company. The destination was Hot Wells, where awaited the medicos a barbecue to delight the most critical epicurean, "free beer and free baths." The best of fellowship prevailed and several hours were passed most pleasantly indeed.

SECOND DAY—WEDNESDAY, APRIL 29TH.

Dr. Red, president, called the Association to order and stated that 9 o'clock was the hour set apart by the program for the transaction of important executive business.

The president then called attention to the fact that the examining board would expire with this meeting and that eighteen names should be presented to the Governor. Action was deferred until tomorrow.

Dr. M. M. Smith, secretary of the judicial council, reported the admission of the following new members: Drs. E. B. Stokes, Crockett; W. M. Wolff, San Antonio; W. J. McGee, Groveton; B. E. Whitte, Shelby; E. L. Clough, Beaumont; T. Buerhring, Nordheim; D. G. Wilburn, Runge; W. K. Curtis, Midland; J. D. Yates, Kirbyville; W. L. Crosthwait, Holland; T. W. Moore, LaGrange; H. B. Combs, Bastrop; J. H. Eastland, Waco; W. G. Jameson, Palestine; T. C. Brassel, Cash; B. E. Knolle, Industry; W. H. Allen, Marlin.

DISCUSSION OF SURGERY.

The section on surgery was called and Dr. William Keiller occupied the chair. In the absence of Dr. Gunby, secretary of this section, Dr. Keiller called Dr. H. W. Crouse to the secretary's desk.

Dr. William Keiller read the chairman's address entitled "The Relation of Anatomy to Surgery."

"A Case of Abdominal Suppuration of Obscure Origin," by Dr. A. L. Hatchcock, Palestine, was read by title and referred to the Publishing Committee.

The next paper on the program was "The Anatomical Basis of Scientific Amputation," by Dr. J. E. Thompson, of Galveston. Dr. Thompson not being present, Dr. Keiller announced that he had Dr. Thompson's paper and illustrations in his possession and asked the pleasure of the section. On motion, Dr. Keiller read and illustrated Dr. Thompson's paper.

"Gross and Misroscopic Anatomy of the Vermiform Appendix and its Bearing on Appendicitis," by Dr. H. B. Dechard, of Galveston, was read by the chairman, Dr. Keiller, and was profusely illustrated.

Dr. Shropshire moved that discussion on all papers in the symposium on appendicitis be postponed until all papers be read. Carried.

Dr. H. A. Barr, of Beaumont, read a paper entitled "Appendicitis, with Report of Cases."

COUNCILLORS SELECTED.

The following list of councillors was then read by President Red:

First District—S. T. Turner, El Paso.

Second District—P. C. Coleman, Colorado.

Third District—D. R. Fly, Amarillo.

Fourth District—C. M. Alexander, Coleman.

Fifth District—W. B. Russ, San Antonio.

Sixth District—A. E. Spohn, Corpus Christi.

Seventh District—T. J. Bennett, Austin.

Eighth District—Walter Shropshire, Yoakum.

Ninth District—J. T. Moore, Galveston.

Tenth District—B. F. Calhoun, Beaumont.

Eleventh District—S. R. Burroughs, Buffalo.

Twelfth District—W. R. Blailock, McGregor.

Thirteenth District—J. H. McCracken, Mineral Wells.

Fourteenth District—C. E. Cantrell, Greenville.

Fifteenth District—H. Taylor, Marshall.

Dr. F. P. Miller, of Midland, read a paper on "Appendicitis—a Discussion of Its Treatment."

The papers on appendicitis read by Drs. Dechard, Barr and Miller were discussed by Drs. Crouse, Jenkins, Collins, Weaver, Reuss, Nix, Garrett and Keiller.

"Extra-Uterine Pregnancy" was the title of a paper presented and read by Dr. J. S. McCelvey, of Temple. A discussion followed by Drs. Barr, Lancaster, Paine, Weaver, Moore, Frazier, Orr and Ledbetter.

On motion the Association adjourned until 2 p. m.

THE PRESIDENTS MESSAGE.

The following is the annual report submitted by Dr. S. C. Red, president, and reported back to the Association yesterday morning:

*"Fellow Members of the Association:*

"You doubtless recall that four months ago you received a card from me inviting suggestions as to what, in your opinion, would be for the best interests of the Association. A generous response was given to this request and from it I gain a fairly definite idea as to what the members want.

"Far and away above all other matters the constitution receives the most attention. Varied and diverse views are expressed concerning it, but with one accord they all sound a note of harmony. Nearly every one says: 'Do nothing that will create disorder in the Association.' With such universal feeling of concession, I feel assured that when the subject comes up for disposal, at this meeting, that no difficulty will be found in coming to a satisfactory conclusion.

"The next most noticed in these replies is really a criticism of the officers. Quite a large number suggested that our Association be conducted on more business-like methods. They seem to think that the session should be conducted according to the program and not to allow one session to encroach upon the other. In response to this I wish to say that it shall be my earnest endeavor to conduct the affairs of this meeting 'strictly according to business methods.' If it is conducted too strictly I invite suggestions. You may expect then to have the session called exactly on time, even if there is but one member present. The by-laws will be diligently enforced, so do not think it is anything personal if you are called down.

"The next subject, in point of numbers, claiming the attention of my correspondents was advice of various kinds concerning politics in this Association. The most of them discussing the subject wanted all politics disposed of by a house of delegates, as provided for in the new constitution. Some two or three did not think the members wanted to be disturbed by political affairs, having come for scientific work, and that by leaving such matters alone they would see them attended to.

"Quite a number that did me the honor to reply had no suggestion to make. They were either indifferent or well satisfied with present conditions.

"The cost of membership, in the opinion of some, is too high. Others think we should have a death benefit as in some secret orders. Some give the Osteopaths and Masseurs a rap. Then again quarrantine, educational and health matters are discussed. Amendments to our present State laws, occupation tax and membership are subjects touched upon. Some few want a journal run by the Association. Others want more attention paid to social functions, assigning as a reason that part of the cause for many attending is the opportunity to visit such affairs. One correspondent suggests a clinic at each meeting by some eminent member of the profession. Another suggests that a bureau of information be furnished by the Committee of Arrangement. This would contribute much to the comfort of the members and should be adopted as the custom of each meeting.

"The above by no means covers all the suggestions offered, but gives a general view of the trend of most of them. It simply goes to show that in an Association like this there are many wants to supply. Most of them come for various reasons, political excitement, social functions, a rest, pleasant companions, new scenes and a general good time. No single one gets all he wants, but there is enough of every kind to be a drawing card for all, even to the big city doctor, for sometimes he is there with 'an ax to grind.'

"Now possibly I might interest you for a moment in what may foster very materially the attendance at our meetings, viz.: Form a legion of honor by giving to each member that has attended ten consecutive meetings a badge or medal of appropriate design. The medal could be selected by a committee of three appointed by the president to report at the next meeting. The attendance of the members could be determined by the registration books kept at each meeting that now doubtless are in the hands of the proper officers. If they have not been carefully preserved the date of counting should begin from that time at which the attendance could be definitely determined by the secretary, treasurer and president, acting as a committee. There are those who feel disposed to laugh at decorations of this kind, but men in all times have been pleased with such, and those of our profession that are members of the A. M. A. are proud to wear its buttons in the lapel of their coats. And I doubt not but that some such procedure would act in like manner in this instance.

"There is still another subject well worth the attention of the Association, viz.: a permanent home. There are several cogent reasons why such a move should not be made, the most important of which would be a loss in a great measure of the social features of the meetings. It is but natural to suppose that where the body would meet permanently it would be quite a tax on the city to furnish annually such receptions as we have been accustomed to under the present arrangement. Neither could we expect to secure the local membership obtained by meeting from place to place. There are some advantages, however, in a home that can not be received otherwise, viz.: the opportunity to establish a pathological exhibit, the securing of a medical library, the construction of facilities for holding clinics by eminent members of the profession. Heretofore, although recommended time and again, the Association has seen fit not to establish a journal. This would then in a measure show to the average member some tangible evidence of return for annual dues. Several plans might be suggested as to how this matter could be worked out. One of which could be to set aside a certain per cent of the annual dues, provided they are kept on present standard, until they reach such a sum as to warrant the construction of a building at some central city, where ample facilities in the way of transportation and hotel accommodations exist. There are other and probably better methods that would suggest themselves than this. It is open to discussion. I leave the subject with you and bespeak for it your attentive consideration.

"The president further thinks that it would be desirable for this Association to assume all costs of defending its members against malpractice suits, that is, suits where the ground for action arose during the membership of the party sued. The costs of such suits could very readily be borne by this Association and the security engendered thereby would in a great measure go to cement the union of its members.

The committee, Drs. Smith, Osborne and Hudson, to whom the president's message was submitted, reported favorably, with the exception of the recommendation to establish a State journal and to assume all costs of defending members of the Association against malpractice suits. The report was adopted by the Association, with the exception of the portion referring to a journal of the Association, which, on motion of Dr. H. A. West, was stricken out.

REPORT OF SECRETARY.

The annual report of the secretary, Dr. H. A. West, of Galveston, shows succinctly the following condition of the Association:

On the roll transactions for 1902—Honorary, 22; ordinary, 366. Total, 388. Died, 4. Total now on roll, 344.

Deaths since last report:

Dr. W. A. Adams, Fort Worth, died October 15, 1902.

Dr. A. B. Gardner, Bellville, died October 22, 1902.

Dr. T. C. Osborn, Cleburne (honorary member), died September, 1902.

Dr. B. F. Eads, Marshall, died February 1, 1903.

This report received favorable consideration of committee and reported back to the Association yesterday morning.

NEW CONSTITUTION ADOPTED.

Dr. M. M. Smith offered the following report from the provisional house of delegates on new constitution and by-laws:

*To the President and Members of the State Medical Association:*

We, your Committee of House of Delegates, met on the afternoon of April 28, and upon roll call found delegates representing the medical societies of the State as follows:

Austin District Medical Society, Dr. F. E. Daniel, Austin.

Austin Academy of Medicine, Dr. M. M. Smith, Austin.

Brazos Valley Medical Association, Dr. I. P. Sessions, Rockdale.

Central Texas Medical Association, Dr. J. M. Frazier, Belton.

Comanche County Medical Society, Dr. T. P. Weaver, Del Rio.

Cooke County Medical Society, Dr. F. D. Garrett, Gainesville.

East Texas Medical Society, Dr. E. M. Link, Palestine.

Harrison County Medical Society, Dr. O. M. Heartsell.

Houston Medical Society, Dr. J. D. Osborn, Cleburne.

Kaufman County Medical Society, Dr. James Orr.

Lavaca County Medical Society, Dr. Walter Shropshire, Yoakum.

North Texas Medical Society, Dr. C. E. Cantrell, Greenville.

Practitioners' Society of Dallas, Dr. S. E. Milliken, Dallas.

Rogers Medical Society, Dr. G. T. Thomas.

San Antonio Medical Society, Dr. Boyd Cornick, San Angelo.

Smith County Medical Society, Dr. T. J. Bell, Tyler.

South Texas Medical Society, Dr. J. W. Scott, Houston.

West Texas Medical Society, Dr. W. B. Russ, San Antonio.

Williamson County Medical Society, Dr. J. C. Anderson, Granger.

After discussing the plan of reorganization recommended by the American Medical Association, and inviting the honorable representative of that body, Dr. J. N. McCormack, to explain certain points which were under discussion, your House of Delegates beg leave to make the following report, and recommend its adoption by this Association:

(Here follows the new constitution and by-laws, which is the same as that prepared by the American Medical Association, and which is now in successful operation in the majority of the States of the Union, and which is quickly being adopted by those not yet in line.)

At the end of the report of the House of Delegates the following resolution was incorporated:

*Resolved,* That the constitution and by-laws just adopted shall go into effect upon the adjournment of this annual meeting, except that the outgoing president shall appoint the council provided for in it at his earliest convenience to serve for one year, or until the House of Delegates elects a council in 1904, and that such council shall organize, divide the State into fifteen councillor districts, map out and arrange its organization work for the coming year, and report its plan to this Association before final adjournment.

*Resolved,* That the secretary be authorized to have printed 5,000 copies each of the constitution and by-laws for the State and county societies, for distribution as soon as practicable to the members of this Association, and from time to time to such other physicians as may be indicated by the respective councillors, and that he also be authorized to provide such stationery and blank forms as may be necessary to put the new plan into operation. It is also provided that the secretary's salary remain for the next year the same as at present and that he be authorized to employ a stenographer.

*Resolved,* That the president and secretary are hereby authorized to issue charters to county societies during the year upon the approval of the executive committee of council; and further, be it

*Resolved,* That new members accepted by county societies after the adjournment of this annual meeting who pay their dues to the State Association in advance shall be credited for such dues as for the years 1903 and 1904, practically obtaining membership for two years for one fee, but that this shall not apply to the dues to the county society for the current year.

S. C. RED, President.
M. M. SMITH, Secretary.

The report was unanimously adopted by the Association.

### AFTERNOON SESSION.

At 2 o'clock the Association was called to order by President Red.

The section on medical jurisprudence was called and Dr. J. R. Nichols, of Terrell, was called to occupy the chair. Dr. Nichols read his annual address as chairman.

Dr. Marvin L. Graves presented a paper entitled "Clinical Notes on Mental Diseases." The subject was discussed by Drs. Paine, McGregor and Guinn.

"Degenerative Stigmata," was the title of a paper read and illustrated by Dr. G. H. Moody, of San Antonio. This paper created a profound impression.

"Expert Witness in Court," was the title of a paper sent by Dr. J. C. Carleton, of Bonham, and on motion the paper was read by the secretary of the section and referred to the Publishing Committee.

Dr. Robert B. Sellers, of Comanche, sent a paper entitled "The Plea of Insanity in Our Courts." The paper was read by caption and referred to the Publishing Committee.

A recess was taken for fifteen minutes, and at the end of the recess President Red called the Association to order and announced a memorial service to the memory of members of the Association departed since the last meeting was held. A number of heart-touching talks were made by various members.

The Association then adjourned until evening.

EVENING SESSION.

The Association convened at 8 o'clock.

The section on State medicine was taken up and an address delivered by Dr. Frank Paschal, chairman, of San Antonio.

Other addresses delivered during the evening were as follows:

"The Tuberculosis Test in the Diagnosis of Tuberculosis in Man," Dr. Boyd Cornick, San Angelo.

"The Prevention of Tuberculosis from the Legal Standpoint," Hon. V. B. Proctor, Cuero.

"The Climatology of Fort Davis," Dr. W. T. Jones, Fort Davis.

"The Public Control of Tuberculosis," Dr. W. S. Carter, Galveston.

"The Adaptability of the Climate of Western Texas for the Treatment of Pulmonary Tuberculosis," Dr. C. H. Wilkinson, Galveston.

"The Race Question as Seen by Physicians, with Especial Reference to the White and Negro Races," Dr. James Orr, Terrell.

A STEREOPTICON EXHIBIT.

The above closed the session for the day and following Dr. Stiles, Zoologist of the United States Public Health and Marine Hospital Service, gave a stereopticon talk on the subject of "Hookworm Disease." This is the malady which was discussed so much by the newspapers of the country a few months ago under the sensational title of the "Lazy Disease," and which Dr. Stiles holds accountable for the condition of the "poor white trash" of the sand districts of the South. Although the disease has been known in the old world for 3500 years, it was not known to exist in this country until last fall, when Dr. Stiles proved that the so-called malaria of sand districts was almost entirely hookworm disease, while true malaria occurred chiefly in the clay districts. Malaria is caused by a minute parasite, which lives in the blood, while hookworm disease is caused by a parasite one-half an inch long, which lives in the intestine. Patients with hookworm disease have abnormal appetites, and the "clay eaters," "sand lappers" and "pickle eaters" represent the extreme stage of the malady. Several cases of this affection have been found in Texas, and Dr. Stiles stated that it was undoubtedly to be found among the Mexicans who come here to work.

Dr. G. W. Cole, of Springfield, Mo., Chief Surgeon of the Frisco system, was a guest of the Association.

The proceedings of the Association were reported in shorthand by Dr. Ella Devlin, of Galveston. This lady is most popular with the physicians, and is an expert on stenographic work of this kind.

THIRD DAY—THURSDAY, APRIL 30.

The Nominating Committee, composed of one delegate from each affiliated society, reported the following

OFFICERS FOR CURRENT YEAR.

President, Dr. Frank Paschal, San Antonio; First Vice-President, Dr. F. E. Daniel, Austin; Second Vice-President, Dr. A. S. Hathcock, Palestine; Third Vice-President, Dr. Joe Becton, Greenville; Trustees, Drs. F. D. Thompson, Fort Worth; J. D. Osborn, Cleburne; Walter Shropshire, Yoakum; J. B. Shelmire, Dallas; B. F. Kingsley, San Antonio; Orator, H. A. Barr, Beaumont.

These gentlemen assumed their duties immediately after election, and Dr. Paschal presided over the deliberations of the Association in the morning.

BOARD OF EXAMINERS.

The Nominating Committee selected the names of eighteen members of the Association to be submitted to the Governor. From this selection of eighteen the Governor will appoint nine, which will constitute the State Board of Medical Examiners. Those named by the committee are as follows:

Drs. Jenkins, Daingerfield; E. E. Guinn, Jacksonville; Bell, Tyler; Wilson, Sherman; Loggins, Ennis; Nicks, Stone City; Evans. Palestine; Burroughs, Buffalo; Ruess, Cuero; Smith, Austin; Frazier, Belton; Osborn, Cleburne; Pennington, Justin; Jackson, San

Antonio; Hamilton, Laredo; Turner, El Paso; Scott, Houston, and Jones, of Gonzales. Chairman Committee of Arrangements, Dr. T. J. Bennett, Austin.

CHAIRMAN'S REPORT, COMMITTEE ON STATE BOARD OF HEALTH.

*Dr. S. C. Red, President State Medical Association of Texas, San Antonio.*

SIR: As chairman of your Committee on State Board of Health, I have the honor to report that in compliance with instructions there was prepared an elaborate and comprehensive bill for a State Board of Health and Vital Statistics, of which the State Health Officer was to be ex-officio president and executive officer, as provided for in the Harrison bill, which bill was approved by the Association at our last meeting. There was an irreconcilable difference in your committee on the vital features, those that made the bill acceptable to the Quarantine Department, and the bill was never introduced. We found amongst leading Senators and Representatives a pronounced sentiment against a Board of Health, and an outspoken opposition even on the part of the Committee on Public Health against creating any new offices carrying an appropriation, and we could not have hoped even for a favorable report on such bill. We were confronted with three alternatives: First, to let the matter go over another two years; second, to face again certain defeat; third, to make an effort to secure the passage of a bill creating a Bureau of Vital Statistics and providing for effective sanitation precisely as was provided for in the first bill, except that instead of a State Board of Health, the authority was to be put in the hands of the State Health Officer, as is the case with regard to quarantine, subject to approval of the Governor. This form seemed to offer the only chance for success, and exercising the discretion which the situation demanded, the last mentioned alternative was adopted. The bill was reported favorably in the Senate with the recommendation that it do pass, and was special order for the afternoon of March 6th. It was never acted upon in either house.

In the interest of harmony in the Association I deem it wisest and best to enter into no explanation, but ask that your committee be excused from further consideration of the matter. The two bills are appended, and are a part of this report.

Respectfully submitted,

F. E. DANIEL, Chairman.

At the afternoon session, Dr. Allen J. Smith delivered the address in Pathology, as chairman of the section, and Dr. J. W. McLaughlin read a paper on "Infection and Immunity." A brief executive session was then held, at which a resolution was introduced to the effect that the Marine Hospital Service regard Cuba as a non-infected port, and asking the Southern States to modify their quarantine regulations accordingly. Referred to Drs. West, Jackson, and Daniel. Reported favorably and adopted.

Thursday night at the Grand Opera House, retiring President Red delivered his address. The subject was, "A Medical View of Dowieism, Osteopathy, Temperance and Christian Science." Dr. Marvin L. Graves, superintendent of the Southwest Texas Insane Asylum, then delivered the

ANNUAL ORATION.

The subject was, "The Doctor: His Ideals, His Aims, and His Rewards." I append some extracts from this beautiful address:

"Like Tennyson's maiden at the brook, I come with reluctant feet and still more reluctant mind to the time and place where the tiny brook of your social pleasures and Association diversions and the great river of your professional toils, care and responsibility meet. I come to pour out my libation, humble but heartfelt, to that noble profession of which you constitute types and exemplars, and of which I became enamored when the first throbbings of ambition stirred my boyish fancy, and to which I have been happily wedded for more than a decade.

"Conscious that 'Life is a train of moods like a string of beads, and as we pass through them, they prove to be many-colored lenses, which paint the world their own hue, and each shows only what lies in its focus,' still I am mindful that the medical profession carefully studies each bead as it passes, detects every error of refraction in lens or defect of color, and focuses its scientific searchlight upon every avocation, labor or profession of man with the sole inquiring purpose of determining wherein lies the weal or the woe of the race.

"PORTRAYAL OF THE DOCTOR.

"Then why should we seek to perpetuate fame by the gaudy tinsel of granite monuments or brazen tablets? Tonight I ask your attention as I portray to you 'The Doctor, His Ideals, His Aims, and His Rewards.'

"And what is the doctor? Shall we agree with the Frenchman, whose ready wit characterizes him as a 'man who pours medicine, of which he knows little, into bodies of which he knows less,' or shall we agree with the Christian Scientist, that 'he is simply an error of mortal mind,' indeed the most colossal error that has ever crossed the threshold of human experience? Or—shall we rather conclude, as Emerson did of the gentleman, that 'he is a man of truth, lord of his own actions and expressing that lordship in his behavior, not in any manner dependent and servile either upon persons or opinions or possessions. Beyond this, manhood first, and then gentleness.'

" 'The doctor understands full well—

" 'There is no great and no small
To the soul that maketh all.'

"What are his ideals? Recognizing ideal not merely an existing in idea, but as embodying a just conception of general excellence and a standard or model of perfection—let me submit what the true doctor, and I speak for none other, stands:

"First—For Truth. Truth in the abstract and in the concrete. Truth in theory and in practice. In other words, the conformity of theory with fact. He stands for the eternal verities of human nature against all the abstractions of philosophy and the alluring mysteries of occultism. He believes that honesty is the correct principle, hence he does not advertise.

"Second—He stands for charity. Charity in its broadest signification; true benevolence and genuine beneficence; earnest well-wishing and active well-doing. It is not merely in service, but in dollars and cents, and if we believe with one of our greatest writers that the only real gift is 'a portion of thyself,' I am sure the doctor has laid his highest offering upon the altar of sweet charity. With open hand and heart, he ministers to the afflicted. With sympathy active and alert, day by day and night after night, his ministrations are commanded by the indigent.

"Third—The true doctor stands for patriotism. Devotion to country in war, in peace, in prosperity and adversity. In all the glorious annals of our country's military history he occupies a conspicuous and important place. Never does the tocsin of war sound and the trumpet call to the field of sacrifice and suffering that the doctor does not respond. Amid its most sanguinary conflicts, with hurtling death upon every side, he stands like a granite mountain, a restoring refuge for the wounded. The true and loyal doctor takes no delight in human carnage. He does not indorse the oppression of the weak by the strong, and lends no ready acceptance to the doctrine that 'might makes right.' With the last drop of his unselfish blood he would defend his native land against hostile invasion, or sacrifice himself in the protection of her laws and the preservation of her institutions, but no 'criminal aggression' can enlist his sympathies or command his services.

"Fourth—He stands for science—and believes, with Alexander Pope, that the proper doctrine is:

"'Know thyself, presume not God to scan,
The proper study of mankind is man.'

"For the benefit of the visiting ladies present, I beg to add that the last line has been amended by an appreciative wit to read:

"'The proper study of mankind is woman.'

"And I do not doubt every gallant physician present renders ready allegiance to this amendment. The word science is derived from Latin 'scio,' to know, and literally means, in medical science, the perfect comprehension of every fact and function of the human body and mind, of every environment, drug, medicine or influence that may affect it in health or disease. He must know the intricate details of anatomy, the interesting facts of physiology and the necessary details of pathology. He must understand human nature in the individual and society. He must learn the laws of heredity and the nature of affinity and consanguinity, and by wise counsel, wherever possible, save the State from the perpetuation of the delinquent, dependent and defective aggregors of society by inheritance. He must understand the fundamental and profound facts of sex, and the marriage relation, and be prepared with wisdom and courage to prevent crimes against the race and society by the ignorant and willful. He must also look after all the afflictions of the human body, which God ordained in dignity and glory, but which some of his ministers continually degrade and debase.

"Fifth—He stands for the home and the family. The unit of human society, the opening and inviting door of our National hope and the supreme center and core of our expanding Christianity. Every home in our broad land has a true and loyal friend in the physician. He worships at the altar where the pristine purity of loving motherhood and the inspiring example of noble fatherhood erect their fanes and rear their sons and daughters—'Far from the madding crowd's ignoble strife.'

"The honorable physician could not be induced by any manner of means to prevent his honorable entrance into the world, or condone his neglect or mistreatment afterward. He believes a just conception of and protection by society of the home and family, are the surest guarantees of our liberties as a people and our survival as a nation. And whenever one of our members so far forgets his honor and debases his profession, as to sacrifice innocent childhood to social respectability, he is driven from among us, and becomes an outcast forever.

"Sixth—He stands for duty. The immortal Lee's sublimest word in the English language. Subject, like other men, to disease, suffering and death, who ever heard of a doctor deserting his post when danger threatened or death lurked in every passing breeze?

THE DOCTOR'S REWARD.

"And now in conclusion. What is his reward? I see before me aged men, whose busy years of usefulness and devotion to the profession have brought upon them prematurely the acus senilis of physical decay. I look again, and behold, just stepping out upon life's highway, with buoyant tread and ambitious, hopeful hearts.

"What has been the sustaining recompense of the one through all these fruitful years, and what is to be the guerdon for the promising labors of the other? I will answer:

"First—The consciousness of duty done and real service rendered humanity. When maturing years have sobered ambition and produced reflection of mind on what life has brought—it is a gratifying feeling to know that 'to say well is good, to do well is better.' The doing of a good deed is it own reward, and when we look into history and find the wrecks of ambition we may enjoy a happier frame of mind in contemplating the usefulness of a well-spent professional career.

"Second—With competency, economy, and judgment, the doctor may look forward to financial independence, and when the withering touch of time has palsied his active energies, it will not be

'Over the hills to the poor-house'

with the faithful physician. I need scarcely add here that the accumulation of money requires good business judgment to invest it, as well as professional ability to earn it. Commerce knows no sentiment, and business no sympathy.

"Third—The doctor who merits and attains professional success acquires a personal and social standing for himself and family that can not be bought with money. This, of course, is not the ultima thule of his ambitions and endeavors, but it is a good professional asset, and is a marked reward of the earnest professional life. We live in the atmosphere created by our associations, and with a firm position in educational advancement and in the social esteem of one's fellows, it follows that life's joys are correspondingly increased, and its opportunities for usefulness greatly enhanced.

"Fourth—His own cultured development and character building.

"It has been previously shown that medical men are progressive, aggressive, devoted to science, education and the nobler virtues. It follows, as a necessary corollary, that a distinct hue of character is given by constant association with noble thoughts and lofty ideals.

"Fifth—And finally—A safe and secure position in public esteem and private affection, a monument of love in the homes and hearts of the people. ..

"Henry Clay, in his hour of greatest political and personal disappointment, gave utterance to a noble thought, when he exclaimed: 'I would rather be right than President.' And Jesus, the Nazarene, in his sacrificial life, uttered a still nobler philosophy, in his own self-sacrifice, when it was said of him, 'He went about doing good.' This is approximately true of the genuine medical man, whose mission of mercy and helpfulness ends only in the silence of the tomb.

"And now, my friends, our ideal doctor's busy life is o'er. His ideals ended, his aims accomplished, his rewards enjoyed. His last call is made; his last soothing word is spoken to some heart-stricken mother, as her fresh-falling tears sanctify a new-made grave. His last medical ministration has stayed the ravages of disease and kindled hope, eternal hope in some sorrowing father's heart. His last surgical operation has restored a husband's useful life to an almost widowed home. His last cheery smile has brightened suffering childhood's wan and weary face. His last cordial hand-shake has spurred young manhood into nobler purposes and inspired despairing girlhood into loftier life. And what of him?

"Sir Christopher Wren, the great English architect, who designed St. Paul's cathedral, the Royal Naval Hospital, and the Towers of Westminister Abbey, is said to have ordered inscribed on his tombstone the following Latin sentence:

" 'Si monumentum quaeris, circumspice,' which means, 'If you seek a monument, look around you.' And so, my splendid colleagues, you need no brazen tablets, no granite spires and marble shafts to perpetuate your well-cherished memory and memorialize your deeds of kindness, and when—

" 'Life's duty done,
Life's race run,
Life's crown won,'

"With clear conscience you may—

" 'Wrap the drapery of your couch about you, and lie down to pleasant dreams.'

"And if you seek a monument, only look around you and witness the enduring memory of your labors in the homes and the hearts of the people."

A BRILLIANT RECEPTION.

A large gathering at Turner Hall, Thursday night, in honor of the visiting doctors, was one of the most brilliant affairs ever given in the city. Beautiful palms and potted plants formed the decorations and a vari-colored star blazed above the musicians. Punch was served in the hall from tables handsomely trimmed with flowers.

On the Reception Committee were Mrs. Jackson, Mrs. King, Mrs. Paschal, Mrs. R. E. Moss, Mrs. Amos Graves, Jr., Mrs. Witten Russ, Mrs. B. F. Kingsley. The guests were introduced by Dr. King.

TIME AND PLACE OF 1904 MEETING.

Austin, fourth Tuesday in April; T. J. Bennett, M. D., Chairman Committee of Arrangements.

